# New Insights in the Cytogenetic Practice: Karyotypic Chaos, Non-Clonal Chromosomal Alterations and Chromosomal Instability in Human Cancer and Therapy Response

**DOI:** 10.3390/genes8060155

**Published:** 2017-06-03

**Authors:** Nelson Rangel, Maribel Forero-Castro, Milena Rondón-Lagos

**Affiliations:** 1 Department of Medical Sciences, University of Turin, Turin 10126, Italy; nrangel@unito.it; 2 Doctoral Program in Biomedical Sciences, Universidad del Rosario, Bogotá 11001000, Colombia; 3 Universidad Pedagógica y Tecnológica de Colombia, Tunja 150003, Colombia; maribel.forero@uptc.edu.co

**Keywords:** cancer, chromosomal instability, clonal chromosomal alterations, non-clonal chromosomal alterations, therapy response, therapy resistance

## Abstract

Recently, non-clonal chromosomal alterations previously unappreciated are being proposed to be included in cytogenetic practice. The aim of this inclusion is to obtain a greater understanding of chromosomal instability (CIN) and tumor heterogeneity and their role in cancer evolution and therapy response. Although several genetic assays have allowed the evaluation of the variation in a population of cancer cells, these assays do not provide information at the level of individual cells, therefore limiting the information of the genomic diversity within tumors (heterogeneity). The karyotype is one of the few available cytogenetic techniques that allow us not only to identify the chromosomal alterations present within a single cell, but also allows us to profile both clonal (CCA) and non-clonal chromosomal alterations (NCCAs). A greater understanding of CIN and tumor heterogeneity in cancer could not only improve existing therapeutic regimens but could also be used as targets for the design of new therapeutic approaches. In this review we indicate the importance and significance of karyotypic chaos, NCCAs and CIN in the prognosis of human cancers.

## 1. Introduction

**Figure 1:**
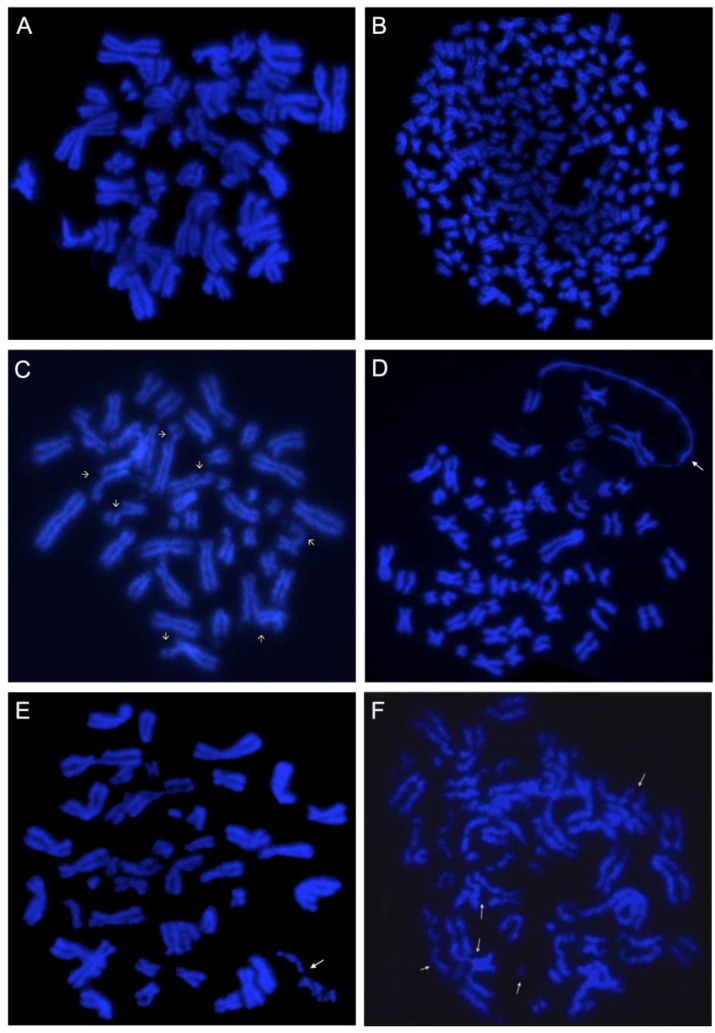
Examples of chromosomal chaos observed in cancer. (**A**) DAPI image of a metaphase with numerical alterations. In these cells, the absence of cell division leads to endoreduplication, a form of nuclear polyploidization that results in multiple uniform changes connected at the centromere. (**B**) DAPI image of a metaphase with polyploidy. In these cells, the chromosome number is greater than 46. (**C**) DAPI image of a metaphase with structural chaos, where chromatid breakage and single sister chromatids are visible (indicated by arrows). (**D**) DAPI image of a metaphase showing a defective mitotic figure. In this metaphase it is possible to observe the co-existence of condensed chromosomes and undercondensed chromatin fibers (indicated by the arrow) within one mitotic figure. (**E**) DAPI image of a metaphase showing early stage C-Frag where most chromosomes are intact. The chromosome being degraded is denoted by the arrow. (**F**) DAPI image of a metaphase with chromosome breakage and several large-scale chromosome fusions. Broken and fused chromosomes can be clearly seen (indicated by arrows).

The term “karyotypic chaos”, also known as “chromosome chaos” or “genome chaos”, is used to refer to the process whereby highly altered, chaotic genomes are formed and the dynamics of continuous change that leads to their formation [1]. Karyotypic chaos has been observed both in experimental systems and clinical samples, singularly in cancers with elevated chromosomal instability (CIN) [2,3,4,5] and drug resistant models [6]. In addition, it has been reported as a common dynamic contributor to cancer macroevolution and progression [1,2,3,5,7]. The types of chromosomal aberrations of a chaotic karyotype are various and include numerical chromosomal alterations (NCAs) (Figure 1A,B), structural chromosomal alterations (SCAs) or a combination of both. SCAs include multiple subtypes such as “chromothripsis” and “chromoplexy”, as well as gene amplifications, deletions, translocations, dicentric chromosomes, inversions and duplications, among others [1,8,9,10], while NCAs include gain or loss of whole chromosomes. Often, many of the above chromosomal alterations are non-recurrent abnormalities (NCCAs) and since these changes are not clonal, they are not taken into account in the cytogenetic analysis, therefore limiting the possibility of finding additional information about both CIN and genomic diversity (heterogeneity) within tumors.

## 2. Clonal and Non-Clonal Chromosome Alterations (NCCAs)

### 2.1. Clonal Chromosome Alterations—CCAs

Usually, cytogenetic analysis has focused on identifying clonal chromosomal alterations (CCAs), specifically those related to specific diseases, while non-recurrent abnormalities have been largely ignored since these are considered as insignificant genetic “noise” and nonessential to clinical applications.

CCAs are defined as a given chromosome aberration which can be detected at least twice within 20 to 40 randomly examined mitotic figures [11] (range of occurrence greater than 30%). CCAs are characterized by the presence of a population of cells, derived from a single abnormal cell, which generally tends to expand and alter (suppress or replace) the growth and development of normal cells. Karyotypic signatures that display recurrent genetic aberrations (CCAs) have been found in many types of tumors and have emerged as prognostic and predictive markers in both hematological cancers and in some types of solid tumors. Moreover, the identification of such cytogenetic abnormalities has increased our knowledge about the mechanisms that lead to tumor development and, most importantly, has led to the development of therapies targeting a specific CCA. For instance, *PTEN*, *ERBB2*, and *ESR1* genes are some of the most specific drug targets used in the cancer treatment, as they undergo genetic changes that lead to the altered expression of their proteins. The major CCAs, altered genes and target therapy of both solid and hematological tumors are summarized in the Table 1.

### 2.2. Non-Clonal Chromosome Alterations (NCCAs)

NCCAs are defined as non-recurrent chromosomal alterations present at a frequency of less than 4% among 50–100 mitotic figures [45]. However, it is important to note that the term “non-clonal” is used to discriminate the clonal karyotypes rather than refering to cells not derived from a common ancestor [46]. It is important to indicate that although NCCAs are still considered by many to be an in vitro culture artifact, the evidence obtained by genome sequencing studies, which indicate the prevalence of massive chaotic genome changes, demonstrated that this is unlikely to be the case. Moreover, Liu et al. [1], using sequencing studies in both in vitro and in vivo (tumors) models, found that complex and heterogeneous end products of tumor evolution reflect each other. However, some NCCAs, such as chromosomal breaks, chromosomal fragmentation and condensation defects, may also be induced either by chemotherapeutic agents or when cells are cultured under conditions that delay DNA replication for diagnostic purposes (fragile X syndrome) [47]. For instance, Heng et al. [48], by the incorporation of 5-Azacytidine (5-aza-C) and 5-azadeoxycytidine (5-az-dC) into late-replicating DNA, induced mitotic chromosome fragmentation and condensation defects. This incorporation can inhibit condensation in mammalian constitutive heterochromatin (human chromosomes 1, 9, 16 and Y) and facultative heterochromatin (inactive X). Further, marked increases of NCCAs have also been observed in lymphocytes from children exposed to antitumoral regimens (radiation or chemotherapy) [49], which have been linked to disease progression. Taking into account the above, the frequencies of NCCAs can be used as a reliable index to measure both internal instability and drug-induced instability of a given cell population or cell line.

NCCAs can be classified into numerical and structural types. Structural NCCAs include chromatid breakage (Figure 1C), single sister chromatids (Figure 1C), Defective Mitotic Figures (DMFs) (Figure 1D), Chromosome Fragmentation (C-Frag) (Figure 1E), Large-Scale Chromosome Fusion (LSCF) (Figure 1F), Chromoplexy and Chromothripsis, among others. Although the above alterations are scarcely reported and frequently ignored, their inclusion within the cytogenetic practice must be considered, as these could provide further information about CIN, genome heterogeneity and cancer evolution.

#### 2.2.1. Chromosomal Breakage

Chromosomal breakage is a type of chromosomal aberration involving DNA breaks (Figure 1C). Chromosome breakage can lead to chromosomal rearrangements as translocations, inversions, dicentric chromosomes, deletions and duplications. These types of alterations are characterized by a defect in DNA repair mechanisms or genetic instability. However, it is important to highlight that double-strand DNA breaks can also be induced by carcinogenic agents, such as reactive oxygen species and radiation. Interestingly, it has been indicated that the number of chromosomal breakpoints can be used to predict the outcome of HER2-negative luminal invasive breast carcinomas in the early stages, where breast cancers with 34 breakpoints or less are indicative of good prognosis [50].

#### 2.2.2. Defective Mitotic Figures

DMFs were initially described as “uncompleted-packing-mitotic figures” [51]. The main features of DMFs are differences in the state of condensation among several chromosomes, leading to coexistence, within a mitotic figure, of condensed metaphase chromosomes and non-condensing chromatin fibers (Figure 1D). In normal mitotic figures, all chromosomes condense at the same rate without the presence of non-condensed chromatin. The possible implications of DMFs on cancer are related to the fact that, in abnormally condensed regions, chromatin fibers may become entangled with each other, which could lead to chromosomal breaks in later stages of the cell cycle when the condensed chromosomes begin to segregate. Chromosomal breaks could therefore lead to subsequent structural alterations (deletions, translocations, inversions), highly implicated in the development and progression of cancer. In fact, DMFs have been detected in cervical and papillary primary tumors and in various cancer cell lines including HeLa (cervical carcinoma cell line), CRL-5824 (small-cell lung carcinoma cell line) and HTB-118 (vulva carcinoma cell line) [52]. The above observations suggest that the chromosomal condensation process is an important factor in cancer.

#### 2.2.3. Chromosome Fragmentation (C-Frag)

C-Frag (Figure 1E) is a form of mitotic cell death where condensed chromosomes are progressively degraded [53,54]. It occurs spontaneously either as a result of certain cellular stresses, such as hereditary genomic instability, or can be induced by treatment with chemotherapeutic agents (doxorubicin and methotrexate) [53]. Therefore, C-Frag is a pathological process that leads to chromosome breakdown, loss of genetic material and cell death [48]. Nonetheless, it is important to note that, if a cell is undergoing C-Frag and does not complete the death process, these fragments can form micronuclei. These micronuclei can lead to the generation of structural chromosomal alterations, either by the action of several repair complexes that “stitch” together such fragments, or can result in double-minute chromosomes if they are retained and replicated. Thus, incomplete C-Frag may potentially lead to genomic instability, which in turn, could increase the complexity of the genome, favoring cancer progression [3,45,55]. Therefore, the degree of C-Frag could also be used as a measurement of induced and spontaneous mitotic death and transient genomic instability.

Morphologically, C-Frag can be grouped into three groups: early fragmentation, midstage fragmentation and final phase of fragmentation, suggesting that C-Frag is a progressive process [53,56]. C-Frag can lead to the induction of chromosomal abnormalities, such as aneuploidies, and to chromosomal chaos [56], both of which have been associated with CIN. In fact, it has been hypothesized that C-Frag is a prerequisite for structural chromosome chaos (chromoplexy), since degraded chromosome fragments could be randomly assembled to form chaotic genomes [5,46]. Further, C-Frag has been associated with various types of cellular stress, including genetic mutations, endoplasmic reticulum (ER) stress, pharmacological treatment and centrosome dysfunction [46]. In fact, it has been suggested that C-Frag could have applications in clinical and basic research, considering that it represents a general response to system stress and can now be used as an easily applied cytogenetic index to measure mitotic cell death and CIN [54,56].

#### 2.2.4. Large Scale Chromosome Fusion

This type of alteration involves chromosomal rearrangements with a high level of complexity, where portions of four or more chromosomes are part of the same chromosome (Figure 1F). Such alterations provide evidence of repeated rupture and fusion events. LSCF can conduce to more complex alterations including amplification, deletion and/or translocation. It is noteworthy that most of these rearrangements are unbalanced (reciprocal translocation is not present) and are not found in all cells within a single cancer [51], thus being NCCAs.

#### 2.2.5. Chromoplexy

The term “chromoplexy” (from the Greek for “chromosome” (*chromo*), and *pleko*, meaning to weave or to braid), has been recently introduced [57] to describe complex genome restructuring including complex structural rearrangements (multiple translocations involving multiple chromosomes). Chromoplexy displays fusion of genomic segments generated by random breakage. Such fusion is apparently mediated by non-homologous end-joining bindings (NHEJ) [58]. The chromosomal rearrangements characteristic of chromoplexy are unclustered and usually include multiple chromosomes. Liu et al. [1] demonstrated that chromoplexy is generated after C-Frag, where C-Frag occurs early in the chromosomal chaos process and is followed by genome re-organization.

#### 2.2.6. Chromothripsis

The term “chromothripsis” (from the Greek for “chromosome” (*chromo*) and “shattering into pieces” (*thripsis*)), has been used to describe one subtype of chaotic genome, with highly rearranged chromosomes affecting one or a small number of chromosomes [59]. Recent reports have indicated that in tumor cells, chromothripsis leads to loss of tumor suppressor genes, deregulation of genes with known cancer links [60] and amplification of oncogenes. Taking into account that chromotripsis affects a large number of genes at a time, it can not only favor the gradual accumulation of mutations but may also quickly stimulate the development or evolution of cancer [61]. However, according to recent reports, although chromotripsis rarely gives rise to clonal fixation in some forms of cancer, because by selective pressure this type of alteration is prone to elimination during clonal outgrowth [62], it has been proposed that those that survive may be in the process of becoming cancerous [63]. Indeed, chromothripsis is thought to represent a driving force of cancer development and progression. For instance, chromothripsis has been associated with poor patient survival, aggressive malignant phenotype [64], rapid disease progression, and early recurrence in many types of cancer [65,66,67,68,69]. The above observations suggest that chromothripsis is a phenomenon of potential relevance not only as a predictive marker but as a therapeutic target in cancer. However, despite the potential use of chromotripsis as a therapeutic target, specific therapeutic options are currently not available [63]. Many mechanisms have been proposed as triggers of chromotripsis, including ionizing radiation acting upon condensed chromosomes [54], telomere erosion and bridge-breakage-fusion cycles [59,60], abortive apoptosis [70], premature chromosome compaction [71], replication stress [60] and pulverization of chromosomes in micronuclei (MN) [72,73].

MN are aberrant nuclear structures resulting from many cell division defects, including errors in DNA replication or repair that generate acentric chromosome fragments [72] and mitotic errors. These mitotic errors generate an abundance of micronuclei that predispose chromosomes to subsequent catastrophic pulverization [74,75]. However, although several studies have been done in this regard, the molecular mechanisms that drive chromothripsis remain unclear.

Although chromothripsis was originally detected in chronic lymphocytic leukemia [23], this phenomenon has also been described in patients with developmental delay and/or cognitive defects [59] and in several types of cancer [76] including breast cancer [65], pediatric medulloblastoma, multiple myeloma, acute lymphoblastic leukemia [77], acute myeloid leukemia, Hodgkin lymphoma, medulloblastoma, neuroblastoma, colorectal cancer and melanoma [66,78]. Moreover, chromothripsis has been observed more frequently in glioblastoma (39%) [79], bone cancers (33%) [80] and lung cancer (21%) [79].

## 3. CCAs, NCCAs and Cancer Evolution

Although the incidence of NCCAs is not widely reported, a high frequency of them can be detected in both tumor samples and lymphoid malignances. Even more, NCCAs have been associated with cancer evolution, tumor progression and poor prognosis in many types of cancer (Table 2); but its implications in cancer evolution are still a topic of debate. For instance, some authors have suggested that although NCCAs are not stable and cannot survive, they provide the necessary genetic variation for macrocellular evolutionary selection. Further, although many chaotic genomes do not immediately undergo cell death, they continually rearrange their genome for a period of weeks after exposure to stress. Such a reorganization process leads to the formation of several new genomes with unique network structures, thus contributing to increased heterogeneity. Only cells that are compatible with the current environment will survive the process and expand clonally [1]. In fact, Liu et al. [1], by treating several cell lines with doxorubicin for 2 h, showed that over 90% of mitotic cells had NCCAs, which persisted in 20% of cells for 2 months after treatment. This highly dynamic change of genome chaos indicates an active fight for survival.

Additionally, it has been proposed that cancer evolution can be characterized as a dynamic relationship between NCCAs and recurrent CCAs, where NCCA-mediated genomic variation plays a dominant role in cancer progression. Indeed, Heng et al. [55] demonstrate that stochastic karyotypic aberrations (NCCAs), rather than sequential recurrent aberrations (CCAs), are the basis for cancer evolution. This model of stochastic interplay of NCCAs and CCAs proposes that a particular CCA (for example CCAa) can be formed stochastically from NCCAs during the cancer evolutionary process and after a certain time period of growth, the CCAa population will then be replaced by the NCCA population, until the next stage where new CCA populations, such as CCAb form and became dominant. Considering the above and, according to Heng et al. [48], it is concluded that NCCAs are the key elements initiating the formation of clonal chromosomal changes (punctuated, discontinuous phase) and that NCCAs provide the basis for various populations of clonal changes (gradual phase) that caused the formation of karyotypical heterogeneity in cancer [55,82,83,84,85]. These claims were also reported by Ye et al. (2009) [46], whom by evaluating five well-characterized in vitro tumor progression models representing various types of cancers, found that the highest level of NCCAs was detected coupled with the strongest tumorigenicity among all analyzed models.

Considering the above observations, the use of NCCAs has been suggested as a biomarker of tumorigenecity [46], since NCCA is a driver of tumor growth [86,87,88,89], is essential for cancer evolution [4,11,90], represents genome level heterogeneity, can be useful as an index for genome instability and seems to be the only shared findings among many cancer types. Furthermore, according to Heng et al. [11], NCCAs represent evolutionary potential by creating new genome systems with altered transcriptomes and phenotypes; so their inclusion in the study of cancer is relevant [3,7,46,84,90,91].

## 4. Chromosomal Instability

CIN, defined as the rate (cell-to-cell variability) of changed karyotypes of a given cell population [7], has been recognized as a hallmark of cancer [85] and as a source of genetic variation favoring tumor adaptation to stressful environments and cytotoxic effects of anti-cancer drugs. CIN can be classified as numerical CIN or structural CIN [92]. Numerical CIN is determined by gain or loss of whole chromosomes (aneuploidy) [93], while structural CIN is determined by gain or loss of fractions of chromosomes. It should be noted that, according to recent reports, the structural CIN is mainly due to the presence of NCCAs [7]. Further, in accordance to the level of NCCAs, CIN can also be classified as stable or unstable. Unstable CIN is present when high levels of NCCAs are detected, while stable CIN is present when specific CCAs are established and are coupled with low frequencies of NCCAs [3].

Although CIN is the most prominent form of genomic instability in solid tumors [91], recent reports have indicated that CIN in itself is not necessarily a common denominator in all cancer cases, since diverse variability degrees of CIN within tumor subtypes can be observed [65]. Moreover, cytogenetic and molecular observations show that solid tumors are characterized by multiclonality, suggesting the existence of a high degree of inter- and intra-tumor heterogeneity, mostly sustained by CIN [91,93,94].

**Figure 2:**
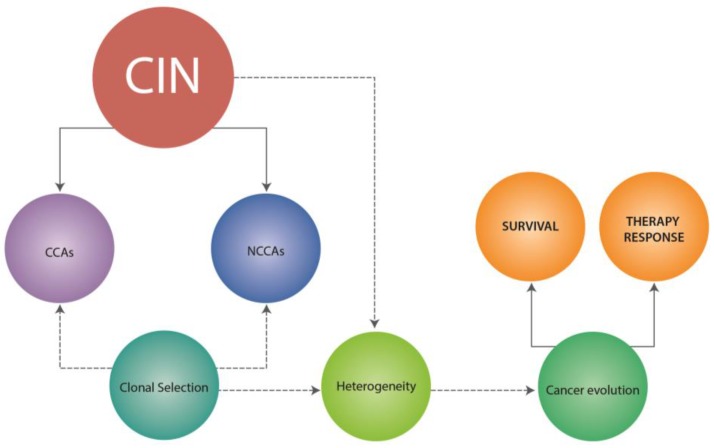
Role of chromosomal instability (CIN) in cancer. CIN is characterized by the presence of both clonal (CCAs) and non-clonal chromosomal alterations (NCCAs). CCAs and NCCAs can conduce to clonal selection and expansion of chromosomal alterations and thus to an overall increase in heterogeneity. Both clonal selection and heterogeneity reflect system instability and drive cancer evolution by increasing population diversity. In addition, CIN and tumor heterogeneity have been linked to tumor cell survival and therapy response.

Accordingly, it is not surprising that CIN and karyotypic heterogeneity have been involved in the somatic cell evolution process, which is the basis of many common and complex diseases such as cancer (Figure 2). Indeed, Heng et al. [3], by comparing the frequency and types of NCCAs and CCAs, noticed that the NCCA/CCA cycle corresponds well with cancer progression.

In addition, CIN and karyotypic heterogeneity has been linked to an increased incidence of metastasis, inferior outcome, advanced stage tumors, cancer progression, increased invasiveness and response to therapy [4,7,95,96,97] (Figure 2).

## 5. NCCAS, CIN and Therapy Response

**Figure 3:**
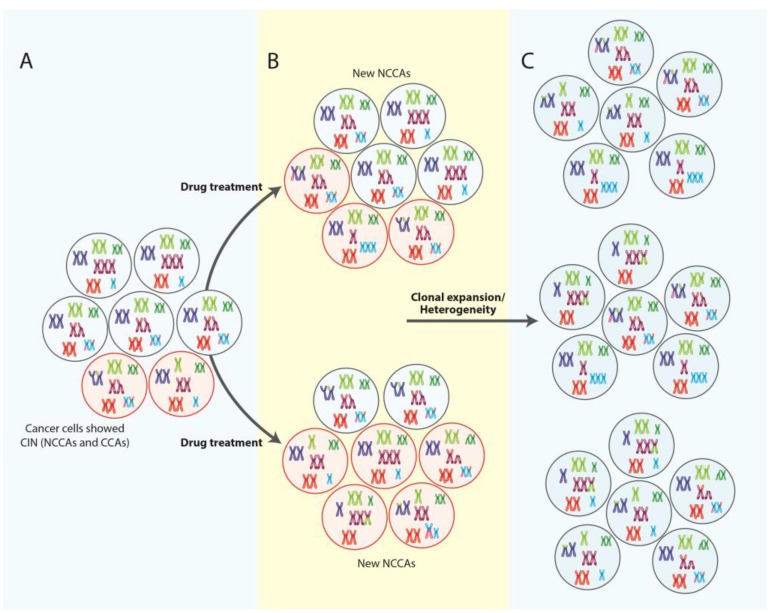
Role of CIN in cancer evolution and therapy response. (**A**) CIN, including both clonal (black circles) and NCCAs (red circles), could impact cell responses to drug treatments. NCCAs can lead to variable drug sensitivity, promoting the survival of a fraction of the tumor cell population. (**B**) The administration of high-dose chemotherapeutics can result in the generation of new NCCAs (red circles), ultimately giving the disease a chance for recovery and resistance. (**C**) CIN can conduce to clonal expansion of NCCAs and thus to an overall increase in heterogeneity, favoring cell survival and therapy resistance. Chromosomes with two colors indicate the presence of structural alterations (translocations).

The importance of NCCAs and CIN in therapy response lies in that these can lead to gene regulatory interactions and varying protein concentrations, both of which could impact cell responses to drug treatments [98]. In this regard, it has been indicated that chromosomal alterations in individual cancer cells (NCCAs) can lead to variable drug sensitivity, promoting the survival of a fraction of the tumor cell population [99] (Figure 2). Further, according to Horne et al. [100], the administration of high-dose chemotherapeutics can also result in the generation of new NCCAs, ultimately giving the disease a chance for recovery and resistance (Figure 3). In fact, we demonstrated that low doses of Tamoxifen (TAM) promotes the production of specific chromosomal abnormalities in breast cancer cell lines, which could contribute to the selection of clones with proliferative advantages, thereby favoring cell survival and therapy resistance [101]. Similar results have been reported by others, where drug treatment (doxorubicin) conduce to clonal expansion of chromosomal alterations and thus to an overall increase in heterogeneity [98] (Figure 3). Indeed, it has been suggested that CIN provides to the tumor cells the heterogeneity necessary to adapt and survive to external pressures including chemotherapeutic agents and radiotherapy [102,103]. Further, numerical CIN (aneuploidy) has also been associated with resistance to immunotherapy. Actually, Davoli et al. [104], by comparing the gene expression profiles of tumors with high and low aneuploidy levels, found that tumors with high levels of aneuploidy correlate with reduced expression of markers for cytotoxic immune cell infiltrates (markers of immune evasion-immune signature), with poor response to immunotherapy and with poorer survival. These results indicate that a high frequency of aneuploidy could be considered a useful marker for predicting response to immunotherapy.

All these observations argue strongly about the profound deleterious effect that drugs may exert on chromosome stability and supports the idea that NCCAs and CIN in cancer are acquired and/or increased during tumor treatment (Figure 3), emphasizing the importance of including them in the study of cancer.

In the last few years, the implications of CIN in the therapy response and in the prevention of tumor relapse [96] have acquired great importance, since high CIN has been associated with the sensitization of cancer cells to therapeutic agents, probably due to excess genotoxicity [105,106]. However, it is important to consider that CIN represents an attractive therapeutic target because it offers the possibility of designing therapies that seek not only to increase such instability but also to suppress it, both of which could be beneficial. In fact, it has been suggested that the nature of therapeutic interventions targeting CIN will depend on the existing levels of chromosome missegregation in the specific tumor, as well as the effect of either decreasing or increasing chromosome missegregation on tumor prognosis [107]. For instance, in tumors in which CIN is associated with poor prognosis, such as Diffuse Large B-Cell Lymphoma (DLBCL) [108], suppression of chromosomal missegregation could reduce the frequency of metastasis and resistance to therapy. Nevertheless, given that extremely elevated rates of CIN also appear to decrease tumor fitness in some cancers [106,108,109,110,111,112], further elevating or stimulating chromosome missegregation rates could be beneficial [113,114,115]. The stimulation of chromosome missegregation has been carried out through the activation of mitotic catastrophe [115] or anaphase catastrophe [114], both of which lead to apoptotic death of tumor cells.

Despite the studies performed so far, therapeutic targeting of CIN in cancer is not yet clear and is still at its preclinical stages [109,116]. Beyond, specific associations between CIN and therapy response have been established in both solid tumors and lymphoid malignances.

### 5.1. Solid Tumors

Breast cancer (BC) represents one of the best models to illustrate the clear relationship between solid tumors and CIN. Cytogenetic characterization of breast tumors has led to the identification of very heterogeneous karyotypes, ranging from near-diploid with few chromosome alterations, to tumors with complex highly-rearranged karyotypes [117]. Indeed, recent studies using single cell genome sequencing identified hundreds of subclonal and de novo mutations that were present at low frequencies (<10%) in the tumor mass, thus confirming the presence of NCCAs and CIN in BC [83]. It is noteworthy that different levels of CIN have been associated with prognosis, survival and therapy response in BC. For instance, estrogen receptor (ER) negative (ER-) and triple-negative breast tumors, with high CIN, have been associated with good prognosis and improved clinical outcome [110,111], while ER-positive (ER+), HER2 and luminal B tumors, with low CIN, have been associated with poor prognosis [82,118]. However, in spite of the above observations, some studies suggest that the correlation between high CIN and improved clinical outcome likely results from the greater impact of genotoxic therapy and not from high levels of CIN per se [112]. In addition, CIN has been associated with multidrug resistance [103], therapy response, poor clinical outcome [106,109,110,111] and patient management, specifically with the choice of chemotherapy regimens in ER positive (ER+) BC [106,119].

In the same way, associations between different levels of CIN and drug response were also observed in HER2 positive (HER2+) BC patients. For instance, HER2+ tumors with distinct patterns of karyotypic complexity (high CIN) were associated with sensitivity to anthracycline and platinum-based therapies [119,120,121,122], whilst tumors with relative chromosomal stability (low CIN) were associated with sensitivity to taxanes [122,123]. In fact, clinical trials have demonstrated improved tumor response rates and additive clinical benefits in HER2+ BC tumors when trastuzumab is combined with anthracycline-containing regimens, or when trastuzumab is combined with taxanes [124].

The above observations confirm the notorious heterogeneous nature of this disease and suggest that assessing CIN in BC subtypes can contribute to the development of personalized therapies [118].

### 5.2. Lymphoid Malignancies

Chromosomal aberrations are the most frequent mutations in lymphoid malignancies [125,126]. Chromosomal alterations in these diseases range from breakpoints, in Diffuse Large B-Cell lymphoma (DLBCL), to a high prevalence of complex chromosomal alterations in Mantle cell lymphoma (MCL), Burkitt lymphoma (BL) and Hodgkin lymphoma (HL) [108,127,128]. In these types of malignancies, important correlations between CIN, prognosis and therapy response have also been observed. Indeed, increased rates of CIN in DLBCL were correlated with substantiate inferior outcome, poor prognosis and tumor relapse after successful treatment [96].

Furthermore, increased CIN induced by cancer treatment was recently reported in Hodgkin lymphoma (HL) patients [108]. HL is a malignant neoplasm affecting the lymphoid system and accounts for ~30–40% of all malignant lymphomas [129]. The therapeutic management of HL patients consists of the application of combined therapies such as MOPP (nitrogen mustard, Oncovin, procarbazine, and prednisone), ABVD/P (adriamycin, bleomycin, vinblastine, and dacarbazine or prednisone), and combinations of these schemes with or without radiotherapy [130]. However, although these therapeutic regimens result in 10 years disease-free survival in 80% of HL patients [131,132], it has been indicated that about 20% of HL survivors develop secondary neoplasms [133]. These secondary neoplasms include leukemia, non-Hodgkin lymphoma and solid tumors [130,134,135]. Paradoxically, it appears that the CIN induced by the treatment of primary malignant tumors (radiotherapy or chemotherapy) was the cause of the onset of a second type of cancer in HL patients [108]. In fact, according to the findings reported by Salas et al. [108], 65% of post-treatment HL patients showed a high frequency of chromosomal breaks and complex chromosomal rearrangements, which are indicative of CIN. It is noteworthy that in HL patients, the majority of complex chromosomal rearrangements observed were NCCAs [108], emphasizing again the importance of their inclusion in the study of cancer.

Currently, several new classes of pharmaceutical agents targeting CIN in malignant lymphoid tumors are being developed. One of these agents includes Kinesin Spindle Protein (KSP) inhibitors. KSP is an ATP hydrolase whose function is related to the regulation of microtubule movement during mitosis and with centrosome separation. Inhibition of KSP leads to cell cycle arrest and cell death [136,137]. Preliminary trials of KSP inhibitors in lymphoid malignancy patients indicate efficacy in refractory Multiple Myeloma (MM) [138], ability to induce disease stabilization in refractory DLBCL and favorable toxicity profile [139], confirming that CIN may be a therapeutic target useful not only to improve the response to therapy but also to reduce side effects in lymphoid malignancies.

## 6. CIN and Radiotherapy

**Figure 4:**
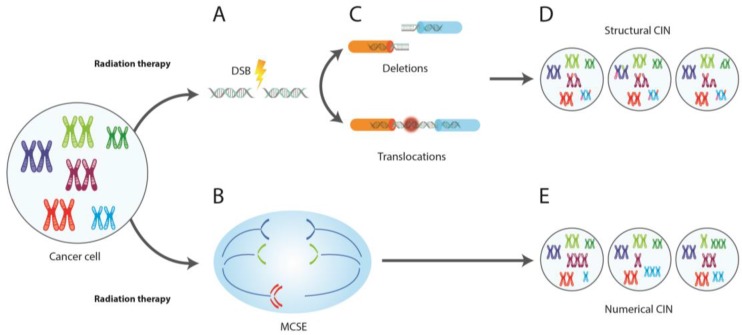
Numerical and structural CIN induced by radiotherapy. Radiation exposure can cause both (**A**) DNA double-strand breaks (DSBs) and (**B**) mitotic chromosome segregation errors (MCSE). While DSBs misrepair can lead to chromosomal rearrangements such as (**C**) deletions and translocations (structural CIN), MCSE can conduce to whole chromosome gains and losses (numerical CIN). Both DSBs and MCSE predispose chromosomes to subsequent (**D**) structural (sub-chromosomal gains, losses and translocations) and (**E**) numerical alterations (monosomies and trisomies), thereby increasing CIN.

Radiation therapy or radiotherapy is an integral modality used in cancer treatment [140]. The lethal effect of ionizing radiation (IR) lies in its ability to induce DNA double-strand breaks (DSBs). This genomic damage leads to reduced cell viability and cell death, due to the decreased ability of tumor cells to repair DSBs. Even more, experimental and clinical evidence indicates that IR exposure during mitosis leads not only to direct DNA breaks but also to numerical chromosomal alterations [75]. IR induces numerical chromosomal alterations by disrupting the process of whole-chromosome segregation during the anaphase. These chromosome segregation errors could not only predispose chromosomes to subsequent alterations, thereby increasing radiation-induced genome damage, but could also impact the viability of irradiated mitotic cells, as the selective suppression of these errors (through destabilization of kinetochore-microtubule stability) leads to a significant increase in mitotic cell resistance to IR. In fact, Bakhoum et al. [75] reported that the suppression of numerical CIN by altering kinetochore-microtubule attachment stability leads to significant tumor radiation resistance, probably by suppressing cell death. The above indicates that exposure to IR induces both numerical and structural CIN (Figure 4).

In the clinical context, the relationship between structural CIN and IR has been widely recognized [141], since it has been observed that unstable tumors with a high frequency of structural CIN tend to respond better to radiation treatment. Nevertheless, it is important to note that sensitivity of cells to IR is not only dependent on the amount of DNA damage that results from IR exposure, but also on pre-existing damage in tumor cells. Interestingly, recent reports have indicated that elevated rates of numerical CIN in rectal adenocarcinoma patients forebode a superior response to chemoradiation therapy. In fact, Zaki et al. [142] reported that rectal adenocarcinoma patients with elevated chromosome missegregation had an enhanced pathological response to chemoradiation therapy. These results suggest that the rate of numerical and structural CIN might independently contribute towards the sensitivity of mitotic cells to IR.

## 7. Conclusions

NCCAs and CIN may play an important role in increasing the number of phenotypes of cancer cells, allowing them to survive selective pressures in the tumor microenvironment, including chemotherapy and radiotherapy. Given that cancer is characterized by unstable karyotypes, to define the level of CIN (CCAs and NCCAs) and heterogeneity could contribute not only to increase our knowledge about cancer but also to identifying the clinical outcome, the most appropriate treatment for patients and novel therapeutic opportunities. Further, the correlations established by the studies conducted to date between NCCAs, CIN and cancer evolution makes their detection clinically relevant. Indeed, NCCAs may therefore be an important but previously unrecognized source of genetic variation.

## Figures and Tables

**Table 1:** Representative examples of clonal chromosomal alterations (CCAs) characteristic of solid and hematological tumors.

CCAs	Altered Genes	Disease	Target Therapy	References
amp(1)(q32.1)	*IKBKE*	Breast cancer	Inhibitor CYT387	Barbie, et al., 2014 [12]
amp(2)(p24.1)	*MYCN*	Neuroblastoma		Yagyu, et al., 2016 [13]
amp(3)(p14.2-p14.1)	*MITF*	Malignant melanoma		Garraway, et al., 2005 [14]
				Kim, et al., 2006 [15]
amp(6)(q25.1)	*ESR1*	Breast cancer	Tamoxifen	Holst, et al., 2007 [16]
				Albertson, et al., 2008 [17]
amp(7)(p12)	*EGFR*	Various cancers	Cetuximab, panitumumab, gefitinib	Sharma, et al., 2007 [18]
amp(17)(q21.1)	*ERBB2*	Various cancers	Trastuzumab, lapatinib	Hudis, et al., 2007 [19]
del(4)(q12q12)	*FIP1L1-PDGFRA*	Myeloid neoplasm associated with eosinophilia	Imatinib	Cools, et al., 2006 [20]
del(5)(q32)	*RPS14*	Myelodysplastic syndrome	Lenalidomide	Ebert, et al., 2008 [21]
del(10)(q23.3)	*PTEN*	Various cancers	Sirolimus	Sansal, et al., 2004 [22]
del(17)(p13.1)	*TP53*	Various cancers		Herrero, et al., 2016 [23]
del(21)(q22.3q22.3)	*TMPRSS2-ERG*	Prostate cancer		Tomlins, et al., 2005 [24]
dup(6)(q22-q23)	*MYB*	Acute lymphoblastic leukemia	MicroRNA-193b-3p	Mets, et al., 2015 [25]
inv(2)(p21p23)	*EML4-ALK*	Non–small-cell lung cancer		Soda, et al., 2007 [26]
inv(10)(q11.2q11.2)	*RET-NCOA4*	Papillary thyroid cancer		Dillon, et al., 2012 [27]
inv(10)(q11.2q21)	*RET-CCDC6*	Papillary thyroid cancer		Dillon, et al., 2012 [27]
inv(16)(p13.11q22.1)	*CBFB-MYH11*	Acute myeloid leukemia		Licht, et al., 2005 [28]
t(1;22)(p13;q13)	*RBM15-MKL1*	Acute megakaryoblastic leukemia		Ma, et al., 2001 [29]
t(2;3)(q12-q14;p25)	*PAX8-PPARG*	Follicular thyroid cancer		McIver, et al., 2004 [30]
t(2;5)(p23;q35)	*ALK-NPM1*	Anaplastic large-cell lymphoma		Mathas, et al., 2009 [31]
t(4;14)(p16.3;q32.33)	*WHSC1-IGHG1*	Multiple myeloma		Bernheim, et al., 2010 [32]
t(5;12)(q31-q32;p13)	*PDGFRB-ETV6*	Myeloid neoplasm associated with eosinophilia	Imatinib	Bain, et al., 2010 [33]
t(8;21)(q22;q22.3)	*RUNX1-RUNX1T1*	Acute myeloid leukemia		Licht, et al., 2005 [28]
t(8;14)(q24.21;q32.33)	*MYC-IGHG1*	Burkitt’s lymphoma		Zech, et al., 1976 [34]
				Taub, et al., 1982 [35]
t(9;22)(q34.1;q11.23)	*BCR-ABL1*	Chronic myeloid leukemia, acute lymphoblastic leukemia, acute myeloid leukemia	Imatinib, dasatinib, nilotinib	Nowell, et al., 2007 [36]
t(9;11)(p22;q23)	*MLL-MLLT3*	Acute myeloid leukemia		Soler, et al., 2008 [37]
t(11;22)(q24.1;q12.2)	*FLI1-EWSR1*	Ewing’s sarcoma		Turc-Carel, et al., 1983 [38]
t(11;14)(q13;q32.33)	*CCND1-IGHG1*	Mantle-cell lymphoma		Al-Kawaaz, et al., 2015 [39]
t(12;15)(p13;q25)	*ETV6-NTRK3*	Various cancers		Seethala, et al., 2017 [40]
t(12;21)(p13;q22.3)	*ETV6-RUNX1*	Acute lymphoblastic leukemia		Uphoff, et al., 1997 [41]
t(12;13)(p13;q12.3)	*ETV6-CDX2*	Acute myeloid leukemia		Chase, et al., 1999 [42]
t(14;18)(q32.33;q21.3)	*IGHG1-BCL2*	Follicular lymphoma		Bakhshi, et al., 1987 [43]
t(15;17)(q22;q21)	*PML-RARA*	Acute promyelocytic leukemia	All-*trans* retinoic acid, arsenic trioxide	Licht, et al., 2005 [28]
t(21;22)(q22.3;q12.2)	*ERG-EWSR1*	Ewing’s sarcoma		Sorensen, et al., 1994 [44]

**Table 2:** Some non-clonal chromosomal alterations (NCCAs) observed in both solid tumors and lymphoid malignances.

Neoplasia	Type of NCCAs	Incidence	Correlated with	References
Primary breast tumors	Chromotripsis	41.4%	Early recurrence, high risk tumors	Przybytkowski, et al., 2014 [65]
Multiple myeloma	Chromotripsis	1.3%	Poor clinical outcome, rapid release	Magrangeas, et al., 2011 [67]
Neuroblastoma	Chromotripsis	18%	Poor prognosis	Molenaar, et al., 2012 [68]
Pediatric cancer	NCCAs	75%	Non indicated	Lopez de Mesa, et al., 2000 [49]
Acute Myeloid Leukemia	Structural NCCAs	7%	Poor prognosis	Niederwieser, et al., 2016 [81]
Cervical, papillary and squamous cell carcinomas	DMFs	NI	NI	Smith, et al., 2001 [52]
Breast cancer, Lipoma	C-Frag	NI	Evolutionary potential	Stevens, et al., 2011 [54]
Brain and hematological malignancies, Leukocytosis.				

DMFs: Defective Mitotic Figures; C-Frag: Chromosomal Fragmentation; NI: Non indicated.
